# Development methods of guidelines and documents with recommendations on physical restraint reduction in nursing homes: a systematic review

**DOI:** 10.1186/s12877-015-0150-9

**Published:** 2015-11-21

**Authors:** Ralph Möhler, Gabriele Meyer

**Affiliations:** School of Nursing Science, Faculty of Health, Witten/Herdecke University, Stockumer Straße 12, D-58453 Witten, Germany; Institute of Health and Nursing Science, Medical Faculty, Martin-Luther-University Halle-Wittenberg, Magdeburger Straße 8, D-06112 Halle (Saale), Germany

**Keywords:** Physical restraint, Nursing homes, Practice recommendations, Evidence-based guidelines, Systematic review

## Abstract

**Background:**

Physical restraint, e.g. bedrails or belts in beds or chairs, are commonly used in nursing homes. However, there have been reports of pronounced differences in the prevalence between different facilities. Guidelines or other documents with recommendations for clinical practice are one approach to overcome centre variation and improve the quality of care. Rigorous development methods are deemed to ensure the validity, clarity and clinical applicability of practice recommendations. This study aims at describing the development methods of documents offering recommendations on physical restraint reduction in geriatric long-term care.

**Methods:**

We performed a systematic search (February 2014) in electronic databases (PubMed, CINAHL, Gerolit, Carelit), the World Wide Web (via google.de) and on the homepages of 34 international scientific or healthcare organisations, using various terms related to documents offering guidance for clinical practice and physical restraints. All German and English language documents with recommendations for clinical practice aimed at reducing physical restraints’ in nursing homes were included. Documents targeting mental health or acute care settings were excluded. Two reviewers independently selected the documents and extracted data, using a self-developed and piloted data extraction form.

**Results:**

We identified 28 documents from Germany, USA, Australia, Switzerland, Canada and UK, published between 2002 and 2014. The documents were developed or published by governmental organisations, nursing or healthcare organisations, non-profit organisation, research institutions and private organisations. Two documents were developed mono-disciplinary (nursing) and eight documents interdisciplinary (including different healthcare professionals, lawyers or other stakeholders). In 18 documents the composition of the development group was not described. Two documents described the methods used for developing the recommendations. In both documents, the recommendations were based on a systematic literature search, critical appraisal of the evidence and developed in a consensus process. Materials or tools supporting the implementation were mentioned in 18 documents.

**Conclusions:**

This review shows that most of the identified documents with recommendations to reduce physical restraints in nursing homes did not adhere to rigorous scientific development methods. Only two documents comprised a systematic literature search and critical appraisal. Guidance aimed to inform clinical practice should rely on transparent and evidence-based methodologically with sound developed recommendations.

**Electronic supplementary material:**

The online version of this article (doi:10.1186/s12877-015-0150-9) contains supplementary material, which is available to authorized users.

## Background

The use of physical restraints (PR) in nursing homes is common practice in numerous countries [1, 2]. The main reasons for using PR are safety issues, e.g. to prevent falls or fall-related injuries; other reasons include the control of challenging behaviours and maintenance of medical therapy [3, 4]. However, there is clear evidence that PR use does not lead to a decrease of falls or fall-related injuries [5–7]. Furthermore, the use of PR is associated with several adverse events, i.e. direct injuries like entrapment or strangulation and indirect events like decreased mobility or reduced psychosocial wellbeing [8–11]. Therefore, restraint-free nursing care is recommended by various international nursing organisations, in numerous statements (e.g. [5, 12, 13]) and scientific publications [14–16].

Epidemiological studies revealed pronounced differences in the prevalence of PR between nursing homes [1, 2, 17]. These differences cannot be explained by characteristics of the residents or the facilities (such as staffing level or staff training) [1, 2]. Evidence-based guidelines can be a powerful tool to reduce inappropriate variation in clinical practice and to increase the quality and efficiency of healthcare [18]. Evidence-based guidelines are systematically developed statements offering recommendations to healthcare professionals to select the best available, effective interventions. The validity, clarity and clinical applicability of the recommendations should be guaranteed by a systematic identification and appraisal of the available evidence and by the development of the recommendations by a multidisciplinary expert panel [19]. The U.S. Institute of Medicine proposes the following standards for trustworthy guideline development: 1) establishing transparency, 2) management of conflict of interest, 3) guideline development group composition, 4) clinical practice guideline – systematic review intersection, 5) establishing evidence foundations for and rating strength of recommendations, 6) articulation of recommendations, 7) external review and 8) updating [20]. When clinical practice guidelines are not available, other guiding documents are needed in order to inform clinical practice. However, the recommendations of such documents are often not based on the evidence but on opinions of experts or the position of an organisation.

In 2009, a German evidence-based guideline [21] was developed, during which a systematic search for guidelines and other documents with recommendations was performed. To this date, no evidence-based guidelines aiming to prevent or reduce physical restraints had been identified [22]. The German evidence-based guideline has recently been updated. The systematic review of guidelines and other documents with recommendations aimed at reducing the use of physical restraints in nursing homes has also been updated.

## Methods

The aim of this systematic document analysis is to describe the development methods used in guidelines and other documents with recommendations aimed at informing clinical practice how to reduce the use of physical restraints in geriatric care.

### Systematic search of documents

A systematic search was performed (Febuary 2014) to identify documents which focus on reducing the use of physical restraints in nursing homes by offering recommendations for clinical practice. Documents were systematically searched in electronic databases (PubMed, CINAHL, Gerolit, Carelit), websites of 34 international scientific or health care organisations (see Additional file 1) and the World Wide Web (via search engines google.de). Inclusion criteria for documents were as follows: offering recommendations for clinical practice to prevent or reduce the use of physical restraints in nursing homes (other settings could also be targeted), published in German or English, and freely available. Documents such as guidelines, policy papers, standards, code of practice, position papers were eligible for inclusion. Documents specifically developed for psychiatric, acute care or hospital settings were excluded.

The following search terms were used: “evidence based, (clinical practice) guideline, recommendations, standards, principles, nursing homes, long-term care, aged, elderly, geriatric care, physical restraints, bedrails and side rails” both in German and English.

### Study selection

Two reviewers independently assessed all titles/abstracts or documents identified by the search for inclusion according to the inclusion criteria. Disagreements were resolved by discussion or, if necessary, referred to a third reviewer.

### Data extraction and analysis

Two reviewers extracted data independently from all included documents using a self-developed data extraction form. The results were checked for accuracy and in the case of disagreement a third reviewer was called in to reach consensus.

The data extraction form was developed on the basis of AGREE [23] and pilot-tested for the first systematic review [22]. For this update, the form was revised according to AGREE 2 [24] and piloted. The data extraction form comprised 34 items covering the following domains: formal criteria (e.g. country of origin, publication date, funding source, authors (individuals and/or organisations)), aim and target group, composition of the development group, development methods of the recommendations, and information and tools for the implementation.

In this systematic review, we focus on the development methods of the identified documents; the content of the recommendations is not part of the analysis.

## Results

**Fig. 1:**
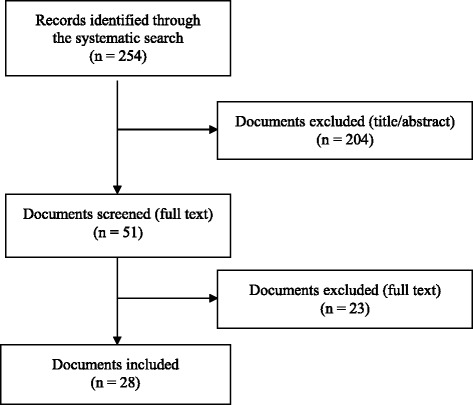
Study selection flow diagram

The search identified a total of 254 documents (see Fig. 1), of which 203 were excluded in the first step. From the 51 documents screened in full text, 28 documents were included in the analysis. The main reasons for exclusion in the second screening were the lack of recommendations and the setting or target group.

### Formal criteria of documents

The documents were developed in Germany (*n* = 11), USA (*n* = 6), Australia (*n* = 5), Switzerland (*n* = 3), Canada (*n* = 2), and UK (*n* = 1) and published between 2002 and 2014. The documents were developed or published by governmental organisations (*n* = 12), nursing or healthcare organisations (*n* = 7), non-profit organisation (e.g. foundations) (*n* = 4), research institutions (e.g. Universities) (*n* = 2), private organisations (*n* = 1) and others (*n* = 2). Information about the authors was available in 13 documents. Seven documents were publicly funded (e.g. by nursing organisations, ministries) and 21 documents did not offer information about the funding.

Scope and purpose were mentioned in 26 and 27 documents respectively. The motivation for the documents’ development were high prevalence rates of PR in nursing homes, risks and adverse effects of PR use, ethical issues like the respect for human rights.

All but one document described one or more target groups: nursing staff (*n* = 22), health care professionals (*n* = 5), relatives (*n* = 4), stakeholders (*n* = 2), or legal guardians (*n* = 1).

Most documents (*n* = 26) comprised a definition of PR, 17 documents described risks or adverse effects of PR use and 20 documents the legal aspects of PR use in the country in which the document was developed.

### Methodological characteristics of documents

Two documents were developed mono-disciplinary (nursing), eight documents interdisciplinary (including different healthcare professionals, lawyer, or other stakeholders), but 18 documents comprised no information about the development group. Three documents aimed to include the interests and perspectives of people suffering from PR. For two documents, patient-representatives were part of the development group (one of these documents additionally performed a literature search on the residents’ perspective) and for one document a patient-representative had the opportunity to comment the draft. Most documents offered no information about conflicts of interest of the development group, or on the role of the funding bodies in the development process.

The description of the methods used to develop the documents’ recommendations was mainly lacking (Table 1). Only two documents described the method used for developing the recommendations (both documents were evidence-based guidelines) and the recommendations of these documents were the only ones based on a systematic literature search and critical appraisal of the available evidence. Both used a consensus process to develop the recommendations but only one of these documents included a grading system for the recommendations.

**Table 1:** Development methods of the included documents^a^

Criterion	No. of documents with fulfilled criterion	
	Yes	No/Unclear
Inclusion of the perspective of patients or residents	4	24
Information on conflicts of interest	2	26
Information on the role of the funding body in the development process	1	27
External review of the draft document	2	26
Piloting	2	26
Development process of the recommendations		
Description of the development methods	2	26
Use of a consensus process	2	26
Recommendations based on a systematic search and critical appraisal of the available evidence	3	25
Use of a grading system for recommendations	1	27
Implementation		
Method for documents’ implementation described	4	24
Availability of implementation materials or tools	18	10
Criteria for evaluating the success of the implementation	4	24
Description of the required resources for the implementation	0	28
Information on barriers or facilitators of the implementation	0	28

^a^A complete list of the included documents as well as the individual ratings for all items is on request available from the authors

A structured approach for the implementation and criteria for evaluating the implementation process were described in four documents. Materials or tools supporting the implementation (e.g. short versions, information leaflets, checklists or flow charts) were available for 18 documents. Information of resources required for the implementation of the documents as well as information on barriers or facilitators of the implementation were lacking in all documents.

## Discussion

This systematic review identified 28 documents with practical recommendations aimed to reduce the use of PR in nursing homes. In most of the documents the development methods were not sufficiently described. For the most part, the recommendations were not based on a systematic literature search or on a critical appraisal of the evidence. Two documents were evidence-based guidelines [12, 20]. In both of these documents the development methods were clearly described and the recommendations were based on both a systematic search and a critical appraisal of the literature.

The reduction of PR was identified as an important issue in the improvement of the quality of nursing [12, 13, 25, 26]. However, the lack of scientific rigour in the development of the recommendations does not reflect the importance of this topic for clinical practice. There is evidence that the implementation of evidence-based guidelines can improve the quality of care [27]. This was confirmed by our own study, which revealed that the implementation of the German evidence-based guideline in nursing homes was effective in reducing PR without increasing adverse effects like falls or fall-related injuries [5]. Currently, the effectiveness and safety of a large-scale implementation of the updated German evidence-based guideline is being investigated in a pragmatic trial [28]. This indicated that the PR use can be reduced by offering recommendations for nursing staff, based on the best available evidence.

Studies investigating the effectiveness of other documents with recommendations for clinical practice are lacking. Evidence-based guidelines using one of the established frameworks for guideline development are expected to ensure valid, transparent, clear and clinically applicable recommendations [20]. Therefore, documents with recommendations on the reduction of PR use in geriatric care should adhere to a methodological framework and rigorous development methods.

This study shows that governmental and professional authorities or initiatives were predominant in initiating the development of the included documents. It remains unclear why these authorities did not initiate the development of evidence-based guidelines. However, the development of a high-quality guideline is a time and cost consuming process. Smaller or local organisations might lack of financial or personal resources. Here, larger networks or cooperation with national agencies like the Institute of Medicine (IOM) or the National Institute for Health and Care Excellence (NICE) might be useful to support the development of guidelines for PR reduction.

We used the criteria of AGREE II [24] to describe the development methods of the included documents. However, most of the included documents were not developed by the use of the methods for evidence-based guidelines. Since the methods recommended for the development of evidence-based guidelines should ensure the validity and clinical relevance of recommendations, the use of these criteria can be judged as a gold standard for developing practice recommendations. Therefore, the criteria used in our analysis reflect the methodological standard and can also be used for other documents offering recommendations for clinical practice.

The reduction of physical restraints has become an important issue for clinical practice in Germany in the last ten years, induced by broad media coverage on this topic. This might be one reason for the large number of documents developed in Germany. Most of the German documents are policy statements made by governmental initiatives or non-profit organisations and often lack a structures dissemination strategy. This fact and the lack of a rigorously developed method might have hampered the national dissemination of most of the German documents. To overcome this gap, several attempts have been made to disseminate the first German evidence-based guideline [21]. The guideline and all corresponding material are freely available; the document has been presented at several scientific conferences; publications addressing nursing and other healthcare professionals as well as informal carers or relatives have been published; and the authors of the guideline have been in contact with other important stakeholders in the field of physical restraints’ reduction in Germany. The same strategy is currently used to support the dissemination and the implementation of the recently updated version of this guideline [26] into clinical practice.

We used a broad search strategy, including databases, relevant organisations and the World Wide Web to identify the documents and included only English and German documents. It is possible that we did not identify all the documents available. However, we identified documents developed in several countries and no further evidence-based guideline was recently listed in the GIN-database. Therefore, chance of missing relevant documents is low.

## Conclusions

This study revealed that the majority of publically available documents with recommendations to reduce the use of PR in geriatric care were not developed by the use of recommended scientific rigorous methods. Since the reduction of PR in geriatric care was identified in many countries as an important indicator for a high quality of care for people with dementia, documents with recommendations for clinical practice should adhere to the methodological standards of evidence-based guidelines.

## Additional file

Additional file 1:
**Websites of German and international scientific or health care organisations included in the search.** (DOCX 23 kb)
