# Supplementary material for: Development methods of guidelines and documents with recommendations on physical restraint reduction in nursing homes: a systematic review
Source: BMC Geriatr. 2015 Nov 21;15:152. doi: 10.1186/s12877-015-0150-9 (PMC4654891; doi:10.1186/s12877-015-0150-9)
Supplement: Additional file 1: — Websites of German and international scientific or health care organisations included in the search. (DOCX 23 kb) [file 12877_2015_150_MOESM1_ESM.docx]

**Additional file - Websites of German and international scientific or health care organisations included in the search**

| **International Organisations** | **Links** |
| --- | --- |
| G-I-N - Guidelines International Network | http://www.g-i-n.net |
| SIGN - Scottish Intercollegiate Guidelines Network | http://www.sign.ac.uk |
| JBI - The Joanna Briggs Institute | http://www.joannabriggs.edu.au |
| AGREE - Appraisal of Guidelines for Research & Evaluation | http://www.agreetrust.org |
| NICE - National Institute for Health and Clinical Excellence | http://www.nice.org.uk |
| RCN - Royal College of Nursing | http://www.rcn.org.uk |
| NIH - National Institutes of Health | http://www.nih.gov |
| IOM - Institute of Medicine | http://www.iom.edu |
| National Guideline Clearinghouse (U.S.) | http://www.guideline.gov |
| JCAHCO - Joint Commission on Accreditation of Healthcare Organizations | http://www.jointcommission.org |
| ANA - American Nurses Association | http://www.nursingworld.org |
| CDC - Centers for Disease Control and Prevention | http://www.cdc.gov |
| GRADE Working Group | http://www.gradeworkinggroup.org |
| FDA - U.S. Food & Drug Administration | http://www.fda.gov |
| AGS - The American Geriatrics Society | http://www.americangeriatrics.org |
| WHO - World Health Organisation | http://www.who.int |
| ICN - International Council of Nurses | http://www.icn.ch |
| **German Organisations** |  |
| AWMF - Arbeitsgemeinschaft der Wissenschaftlichen Medizinischen Fachgesellschaften e.V. (Association of Scientific Medical Societies in Germany) | http://www.awmf.org |
| ÄZQ - Ärztliches Zentrum für Qualität in der Medizin (Agency for Quality in Medicine) | http://www.aezq.de |
| DNQP - Deutsches Netzwerk für Qualitätsentwicklung in der Pflege (German Network for Quality Development in Nursing) | http://www.dnqp.de |
| BUKO - Bundeskonferenz zur Qualitätssicherung im Gesundheits- und Pflegewesen e.V. (Federal Conference for Quality Assurance in Health Care) | http://www.buko-qs.de |
| IQWIG - Institut für Qualität und Wirtschaftlichkeit im Gesundheitswesen (Institute for Quality and Efficiency in Health Care) | http://www.iqwig.de |
| UW/H - Medizinisches Wissensnetzwerk evidence.de der Universität Witten/Herdecke (University Witten/Herdecke - Medical Knowledge Network evidence.de) | http://www.evidence.de |
| MDK - Medizinischer Dienst der Krankenversicherung (Medical Service of the Statutory Health Insurance) | http://www.mdk.de |
| MDS - Medizinischer Dienst des Spitzenverbandes Bund der Krankenkassen e.V. (Medical Service of the Central Association of Health Insurance Funds) | http://www.mds-ev.de |
| GBA - Gemeinsamer Bundesausschuss (Federal Joint Committee) | http://www.g-ba.de |
| BZgA - Bundeszentrale für gesundheitliche Aufklärung (Federal Centre for Health Education) | http://www.bzga.de |
| dip (WISE-Datenbank) (The WISE database of the German Institute of Applied Nursing Research) | http://www.dip.de |
| DBfK - Deutscher Berufsverband für Pflegeberufe (German Nursing Association) | http://www.dbfk.de |
| Deutsches Institut für Menschenrechte (German Institute for Human Rights) | http://www.institut-fuer-menschenrechte.de |
| Deutscher Pflegerat e.V. (German Nursing Board) | http://www.deutscher-pflegerat.de |
| BQS - Institut für Qualität & Patientensicherheit (Institute for Quality & Patient Safety) | http://www.bqs-institut.de |
| Evangelische Hochschule Freiburg (Protestant University of Applied Sciences Freiburg) | http://www.eh-freiburg.de |
| KDA – Kuratorium Deutsche Altershilfe (Board of Trustees of German Assistance for the Elderly) | http://www.kda.de |
